# Exosomes as a biomarker in postoperative complications: a literature review

**DOI:** 10.3389/fcell.2026.1921951

**Published:** 2026-08-18

**Authors:** Zhan Huang, Yi Wang, Wei Li, Zhiqiang Gan, Haiqing Yang, Jiayao Li, Yuan Jiang, Yi Yuan

**Affiliations:** 1 Department of Anesthesiology, Dazhou Integrated TCM and Western Medicine Hospital, Dazhou Second People’s Hospital, Dazhou, China; 2 Department of Rehabilitation Medicine, Clinical Medical College and The First Affiliated Hospital of Chengdu Medical College, Chengdu, Sichuan, China; 3 Department of Orthopedics, Dazhou Integrated TCM and Western Medicine Hospital, Dazhou Second People’s Hospital, Dazhou, China

**Keywords:** biomarker, exosomes, liquid biopsy, postoperative complication, surgery

## Abstract

Surgery remains a common treatment, but it can lead to various potential complications. The incidence of complications after major surgery, in particular, is notably high. Postoperative complications contribute to increased patient morbidity and mortality. Timely and effective identification of these complications is essential for appropriate clinical management and improved patient outcomes. Exosomes are nano-sized membrane vesicles released by a variety of cell types, and play important roles in intercellular communications. Exosomal proteins and nucleic acids are involved in numerous physiological and pathological processes and are increasingly recognized as valuable biomarkers for disease diagnosis. Exosomes are present in all body fluids, which underlies their considerable potential as novel biomarkers in liquid biopsy. Previous research has reported that exosomes can serve as predictive biomarkers for several common postoperative complications, including neurocognitive disorders, allograft rejection, septic shock, atrial fibrillation, and sleep disturbance. These findings suggested that exosomes hold potential for application in the prevention and management of postoperative complications, representing an emerging direction in the investigation of postoperative biomarkers. Therefore, this review highlights the roles of exosomes in postoperative complications and discusses the challenges and opportunities of exosome-based diagnosis for these conditions.

## Graphical Abstract

**Figure:**
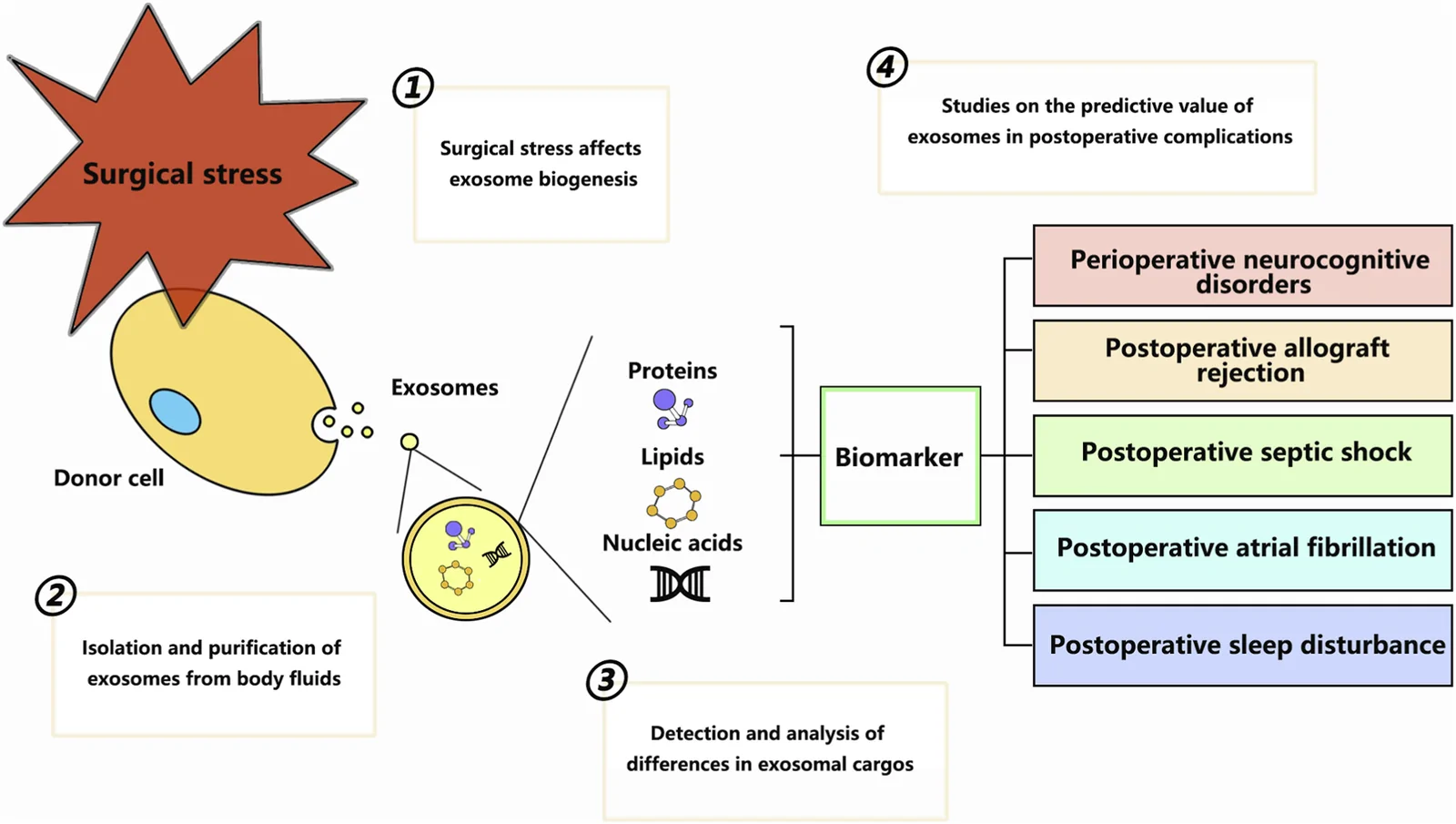
Schematic representation of exosomes acting as biomarkers in postoperative complications.

## 1 Introduction

Postoperative complications are one of the most common surgical adverse events. These complications mainly arise from tissue damage, functional loss, or impairment of other organs and tissues caused by the original disease, surgical trauma, intraoperative bacterial contamination, incisional pain, and restricted limb movement, which often impact surgical outcomes, deteriorate prognosis, prolong the patient’s hospitalization, and cause long-term physical, psychological, and economic burdens. Available data indicate that about 20% of patients develop postoperative complications after surgery (Oiwa et al., 2022; Nye et al., 2024). Identifying early biomarkers is essential for detecting patients at risk of postoperative complications. Current research primarily focuses on blood-based biomarkers due to their accessibility and potential for real-time monitoring. For instance, inflammatory factors, C-reactive protein (CRP), creatinine, S100 calcium-binding protein beta (S100β), and neuron-specific enolase (NSE) have shown diagnostic value in various postoperative complications (Yang et al., 2022; Hassani Ahangar et al., 2025; Mosharaf et al., 2025). In recent years, with a deeper understanding of their unique biochemical and physicochemical properties and biological functions, exosomes have drawn increasing attention as potential biomarkers for disease diagnosis. Exosomes, a subtype of extracellular vesicles (EVs), are released from cells into the extracellular environment. Acting as natural carriers, exosomes facilitate intercellular communication between neighboring or distant cells by transferring their proteins, lipids, and nucleic acids. Both physiological and pathological conditions influence exosomal surface molecules and cargo, presenting a wealth of candidate biomarkers for diagnostic applications (Lin et al., 2015; Jalaludin et al., 2023). Notably, exosomes remain stable in body fluids over extended periods, making them readily obtainable and suitable for real-time monitoring. To date, studies have reported that exosomes can serve as predictive biomarkers for several common postoperative complications, including neurocognitive disorders, allograft rejection, septic shock, atrial fibrillation, and sleep disturbance (El Fekih et al., 2021; Gu and Zhu, 2021; Bai et al., 2022; Zhao et al., 2022; García-Concejo et al., 2025). These findings warrant a summary and discussion to advance the use of exosome-based diagnostics in postoperative complications. The recently published “Minimal information for studies of extracellular vesicles - 2023 (MISEV2023)” defines exosomes as a subtype of small EVs originating from the endosomal system and are released via multivesicular bodies (MVBs) (Welsh et al., 2024). This definition follows from the fact that exosomes are generated through the fusion of MVBs with the plasma membrane via the endosomal pathway, unlike the other two types of EVs, microvesicles and apoptotic bodies, which form by budding directly from the plasma membrane (Wang et al., 2021a). Therefore, in this review, we first briefly describe the characterization, biogenesis, and isolation methods of exosomes before highlighting their roles in postoperative complications. Finally, the challenges and opportunities associated with exosome-based diagnosis of these complications are also discussed.

## 2 Overview of exosomes

### 2.1 Characterization of exosomes

Exosomes are bilayer membrane vesicles sized 30–150 nm, appearing biconcave or cup-shaped under negative staining electron microscopy. Their double-layer membrane structure encloses various components, including proteins, lipids, and nucleic acids (Vafaei et al., 2022). Approximately 4400 proteins have been identified in exosomes (Moeinzadeh et al., 2022). Among them, certain characteristic proteins such as tetraspanins serve as markers that distinguish exosomes from other EVs and are involved in membrane fusion, signaling, and protein trafficking (Andreu and Yáñez-Mó, 2014). The lipid components of exosomes, including glycosphingolipids, sphingomyelins (SM), cholesterol (CHOL), and phosphatidylserine (PS), are essential for exosome membrane stability and structural integrity (Chen et al., 2024). Nucleic acids, including deoxyribonucleic acid (DNA), messenger RNA (mRNA), and microRNA (miRNA), are another key exosomal cargo, influencing phenotypic changes in recipient cells and enabling intercellular communication and epigenetic regulation (Quesenberry et al., 2015; Liu et al., 2024; Phillips and Noble, 2024). In particular, miRNA profiles can reflect the physiological or pathological state of parent cells (Isaac et al., 2021). Analyzing exosomal contents to estimate functional states of the parental cells is the foundation for the development of exosome-based diagnostics (Li et al., 2026). Notably, the biogenesis of exosomes directly determines the complexity and functional diversity of their components.

### 2.2 Biogenesis of exosomes

**FIGURE 1:**
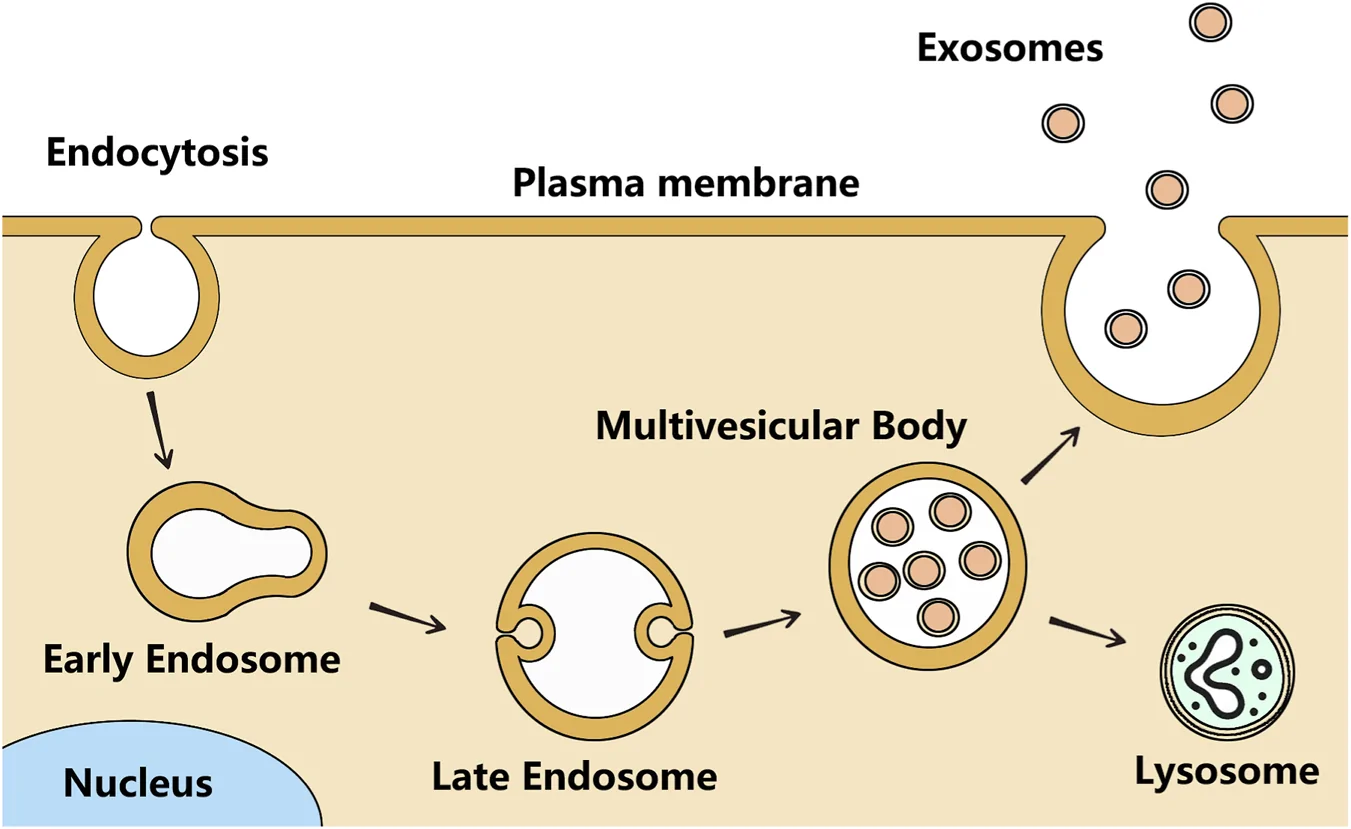
Schematic representation of exosome biogenesis.

Exosome biogenesis is a complex process that involves multiple steps. In brief, it begins with the formation of the early endosome through inward budding of the plasma membrane. During the maturation of early endosomes into late endosomes, the endosome membrane invaginates and buds to generate numerous nanoscale intraluminal vesicles (ILVs), leading to the appearance of multivesicular morphology in late endosomes, which are termed multivesicular bodies (MVBs). Then, MVBs either fuse with lysosomes, leading to degradation of cargo within the ILVs, or fuse with the cell plasma membrane, leading to secretion of ILVs into the extracellular environment as exosomes (Figure 1) (Jiang et al., 2020; Xie et al., 2022; Al-Madhagi, 2024). Multiple pathways and molecules are involved in exosome biogenesis, particularly during exosomal cargo sorting and exosome release. The formation of ILVs is a key process in selecting and loading cargo into exosomes, as the invagination of the endosome membrane encloses proteins, nucleic acids, and lipids within them. This process involves both endosomal sorting complex required for transport (ESCRT)-dependent and ESCRT-independent pathways. The ESCRT complex plays important roles in the ILV generation machinery, which comprises ESCRT-0, -I, -II, -III, and accessory proteins. ESCRT 0 is charged with identifying and sorting cargoes into functional microdomains on the endosomal membranes. ESCRT-I and ESCRT-II promote the budding of the endosomal membrane to form the initial bud. ESCRT-III is charged with cleaving the neck of the bud formed to produce ILVs (Krylova and Feng, 2023). Some proteins have been confirmed to be involved in ESCRT-independent pathways. For example, Tetraspanins, as protein markers of exosomes, are closely associated with cargo sorting because they organize the endosomal membrane into tetraspanin-enriched microdomains (TEMs), which serve as platforms for cargo transport, directing cargo toward MVBs (Perez-Hernandez et al., 2013; Verderio et al., 2018). Ceramides, produced by neutral sphingomyelinase (n-SMase), can promote the budding within raft-based domains on the interior side of the MVB membrane, facilitating RNA loading into MVBs, and their cone-shaped structure may also induce a spontaneous negative curvature to the membrane, thereby promoting the formation of vesicles inside MVBs (Trajkovic et al., 2008; Verderio et al., 2018; Sapoń et al., 2023). In addition, ceramides can be metabolized to produce sphingosine 1-phosphate (S1P), which binds to the S1P receptor on MVBs to regulate exosomal cargo sorting into ILVs and promote the MVB maturation (Kajimoto et al., 2013). Ras-associated binding protein 31 (Rab31) is another key regulator of the ESCRT-independent pathway because it drives the budding of MVB membrane into the lumen to form ILVs and recruits TBC1D2B to inactivate RAB7, thereby suppressing the fusion of MVEs with lysosomes and enabling the secretion of exosomes (Wei et al., 2021). The transport of MVBs to the plasma membrane is a key step in exosomal secretion and requires actin and the microtubule cytoskeleton. Actin provides the driving force for MVBs to move towards the plasma membrane, while the microtubule cytoskeleton provides track support for MVBs’ movement and promotes MVB anchoring and fusion with the plasma membrane. (Yang et al., 2026). Rab GTPase family proteins are the core molecular switches that regulate MVB transport, anchoring, and fusion to the plasma membrane. For example, RAB27a regulates the transfer of MVBs from microtubules to cortical actin, and RAB27b inhibits fusion of MVBs with each other or other vesicles, thereby promoting MVB fusion with the plasma membrane (Arya et al., 2024). Soluble N-ethylmaleimide-sensitive factor attachment protein receptors (SNAREs) proteins also facilitate the fusion of vesicles with the plasma membrane to release exosomes (Hessvik and Llorente, 2018).

### 2.3 Isolation methods

Exosomes, secreted by donor cells into the extracellular environment, are found in various bodily fluids, including blood, urine, saliva, cerebrospinal fluid, alveolar fluid, and breast milk (Hade et al., 2021; Jiang Y. et al., 2022). The components encapsulated in exosomes can reflect the functional status of donor cells under both healthy and diseased conditions, positioning them as potential biomarkers. Notably, choosing the appropriate methods of isolating exosomes from body fluids is a crucial step. Although no standardized isolation protocol has yet been established, six major separation strategies have been summarized and compared in published reviews, including ultracentrifugation, ultrafiltration, immunoaffinity capture, polymer precipitation, size-exclusion chromatography, and microfluidic techniques (Yang et al., 2020; Sani et al., 2024).

Ultracentrifugation: As the gold-standard approach for exosome isolation, it is the most frequently reported strategy (Li et al., 2017). Its principle is that particles with different densities and sizes exhibit different sedimentation rates under centrifugal force. The centrifugal forces for exosome separation typically range from 100,000 to 150,000 × g, enabling the isolation of exosomes from other small particles such as bacteria, viruses, and cellular organelles. Due to its low cost, ease of operation, and absence of complex sample pretreatment, ultracentrifugation is well-suited for large-scale exosome preparation and widely used to isolate exosomes from various body fluids. In practice, ultracentrifugation relies on costly, specialized equipment and centrifuge tubes. High-speed centrifugation can cause mechanical damage and protein aggregation in exosomes. The process typically takes an hour and a half per run and may need to be repeated multiple times. Even so, the obtained exosomes may lack sufficient purity for small-volume diagnostic applications. Some scholars will further use density-gradient ultracentrifugation to enhance the purity of exosomes. The principle of this method is that particles with a particular density would remain suspended in a medium of a similar density after centrifugation. The obtained exosomes are added to the top of the density-gradient medium (e.g., iodixanol or sucrose) in the centrifuge tube (Doyle and Wang, 2019). Then, components with different buoyant densities reach a static position (isopycnic position) in the layer of the same density following prolonged centrifugation. Exosomes distribute across different layers of the medium, allowing separation of subpopulations. Density gradient ultracentrifugation thus yields higher-purity exosome samples and facilitates studies on their subpopulations (Bobrie et al., 2012).

Ultrafiltration: This method uses a filter membrane with a different molecular weight cut-off (MWCO) to isolate exosomes. By adjusting the filter size, researchers can classify specific subsets of exosomes. This process requires less time and no special equipment compared with ultracentrifugation. The available methods include (Lai et al., 2022): a) sequential filtration, which involves multiple rounds of filtration by different MWCOs; b) tandem filtration, which involves combining multiple filters in a single syringe; c) centrifugal ultrafiltration, which involves using centrifugal force that drive the sample through a nanoporous membrane fixed inside a tube for exosome capture; d) tangential flow filtration, which involves a flow stream moves parallel to the membrane surface, and only a portion crosses the membrane by hydrodynamic pressure. Notably, exosome yields are often constrained by filter clogging and shear stress-induced destruction.

Immunoaffinity capture: This method employs antibodies immobilized on surfaces such as magnetic beads, chromatography column resins, multiwell plates, and microfluidic devices to capture exosomes via specific binding between exosomal membrane antigens and the antibodies, achieving high purity and retaining the natural state of the isolated exosomes. Several existing exosome isolation products target transmembrane proteins (e.g., CD9, CD63, and CD81), annexin, and Alix, which have been employed for selective exosome isolation (Kimiz-Gebologlu and Oncel, 2022). Researchers also need to consider limitations such as low yield, high cost, and potential disruption of exosomes through the extra elution steps in this method.

Size-exclusion chromatography: This method uses porous materials, such as dextran (Sephadex), agarose (Sepharose), and polyacrylamide (Sephacryl or BioGel), to separate exosomes by size (Wang et al., 2024). Larger particles that cannot enter the pores are eluted earlier from the gel filtration column. In contrast, smaller ones enter the pores and slow down, thereby increasing exosome yields and even enabling the isolation of an exosome subpopulation. Notably, this method does not compromise exosomal structure or integrity, so the isolated exosomes retain their natural biological activities, making it an ideal method for separating exosomes (Sidhom et al., 2020).

Polymer precipitation: This method uses highly hydrophilic polymers that interact with the water molecules surrounding the exosomes, forming a hydrophobic microenvironment that causes exosome precipitation upon low-speed centrifugation. Polyethylene glycol (PEG), a hydrophilic polymer, is commonly used as the basis for commercial exosome isolation reagents (Ludwig et al., 2018). This method does not require complex equipment, is easy to operate, and offers high yield, contributing to its broader adoption. However, hydrophilic polymers can also precipitate other water-soluble materials, such as lipoproteins and proteins, resulting in a low exosome purity.

Microfluidic technique: This method is based on miniaturized microfluidic apparatuses to separate exosomes by immunoaffinity, size, and density (Hassanpour Tamrin et al., 2021). Its key advantages include rapid exosome extraction from small sample volumes and the ability to perform real-time characterization for *in situ* diagnostics.

## 3 Exosomes and postoperative complications

For identifying and selecting the published studies about exosomes and postoperative complications, a comprehensive literature search was performed across the Web of Science (WoS), PubMed, Scopus, and ScienceDirect databases. All information in this review was obtained from published studies on exosomes and postoperative complications. Search terms included combinations such as [(exosome) or (extracellular vesicle) and (postoperative complications) or (neurocognitive disorders) or (allograft rejection) or (septic shock) or (atrial fibrillation) or (sleep disturbance)]. Duplicate publications, protocols, thesis, and reviews were excluded.

### 3.1 Perioperative neurocognitive disorders (PND)

Perioperative neurocognitive disorders (PND) are an umbrella term for cognitive impairment identified in the preoperative or postoperative period, encompassing pre-existing neurocognitive disorder (NCD), postoperative delirium (POD), postoperative delayed neurocognitive recovery, and postoperative cognitive dysfunction (POCD) (Evered et al., 2018). The underlying pathogenesis of PND remains unclear. The pathophysiological processes of PND involve central synaptic plasticity and function, neuroinflammation, excitotoxicity, and neurotrophic support. Microenvironmental changes in nerve cells following surgery or anesthesia are now considered a key factor in PND pathology (Huang et al., 2022). All neural cells, including neurons, microglia, and astrocytes, release exosomes under physiological and pathological conditions. As carriers for transporting different bioactive molecules between nerve cells in the microenvironment, exosomes have also been confirmed to be involved in the pathological changes of PND. Exosomes can cross the blood-brain barrier (BBB) into the circulatory system, enabling PND assessment by analysis of their contents (e.g., miRNAs or proteins) in blood samples (Das et al., 2025). Therefore, the effect of exosomes in the diagnosis of PND has attracted the keen attention of many scholars.

POCD is characterized by disturbance of consciousness, disordered behavior, aimlessness, and inability to concentrate after surgery, which often occurs in the elderly. Data indicate that 20%–50% of individuals older than 60 years experience these complications after major surgery. The risk rises to as high as 80% among older patients in intensive care units (Raphael et al., 2023; Cho et al., 2024). Although the symptoms of POCD are often transient and reversible, it is associated with a high mortality rate and poor cognitive function, affecting the quality of life. Several non-coding RNAs have been proven to be closely related to the onset of POCD following surgery, especially those that are susceptible to excessive anesthetic drug exposure during the perioperative period (Berger et al., 2015). For example, sevoflurane, a widely used inhalational anesthetic, can contribute to POCD by inducing neuroinflammation, promoting neuronal apoptosis, and inhibiting BDNF expression (Yin et al., 2019; Wang et al., 2021b; Yang et al., 2024). Zhao and colleagues isolated exosomes from the plasma of patients with or without sevoflurane-induced POCD or non-POCD, and performed small RNA sequencing. They identified 184 upregulated and 117 downregulated miRNAs, which were significantly enriched in 3,577 GO terms and 121 KEGG pathways, including Rap1 signaling pathway, ECM-receptor interaction, phospholipase D signaling pathway, PI3K-Akt signaling pathway, and cGMP-PKG signaling pathway. Notably, miR-548-5p was enriched in plasma-derived exosomes associated with POCD. Since BDNF is the target gene of miR-584-5p, POCD-associated exosomes can downregulate brain-derived neurotrophic factor (BDNF) expression and p-TrkB/TrkB levels, and upregulate Caspase 3, leading to apoptosis of HMC3 cells and affecting the development of POCD (Zhao et al., 2022). In animal models, Wei and colleagues found that miR-182-5p was significantly upregulated in POCD rats following inhalation of 2% sevoflurane and clamping of the superior mesenteric artery, and could be transmitted via plasma exosomes. Plasma exosomal miR-182-5p can exacerbate neuroinflammation and cognitive dysfunction by activating the NF-κB pathway and suppressing BDNF expression, while inhibiting them can obviously reduce the learning and memory disorders in POCD rats, suggesting that plasma exosomal miR-182-5p plays a vital role in sevoflurane-induced neuroinflammation and cognitive dysfunction (Wei et al., 2022), and represents a potential biomarker for predicting POCD. This finding may deepen our understanding of the pathogenesis of sevoflurane-induced POCD. The relationship between specific plasma exosomal miRNAs and POCD provides potential biomarkers. In addition to miRNAs, sevoflurane alters the expression of exosomal lncRNAs. Campo and colleagues investigated the expression profiles of lncRNAs in the circulating exosomes of 30 patients undergoing laparoscopic cholecystectomy, before and after anesthesia. Compared with 30 healthy controls, they found overexpression of MALAT1, GAS5, HOTAIR, and BLACAT1 in the serum of subjects after anesthesia. MALAT1 plays a crucial role in the development of POCD, exhibiting a protective effect, and its overexpression can limit oxidative stress and anesthetic-induced neuronal apoptosis by regulating the miR-101-3p/PDCD4 (Programmed Cell Death 4) axis. However, overexpressed GAS5 is associated with worsened cognitive impairment and neuroinflammation. Overexpressed HOTAIR can suppress BDNF and induce cognitive impairment. Overexpressed BLACAT1 promotes reactive oxygen species accumulation and mitochondrial dysfunction through the activity of Caspases 3 and 7, resulting in apoptosis and neuronal degeneration. Campo and colleagues proposed that anesthetics induce certain lncRNAs with neuroprotective effects, and some others are linked to neurological dysfunctions, resulting in a convoluted cascade of lncRNA-mediated counter-responses in POCD processes. The exosomal lncRNAs HOTAIR, GAS5, and BLACAT1 can be considered as potential biomarkers in POCD (Campo et al., 2024). Some scholars have suggested that abnormal levels of circRNA-089763 in plasma exosomes during the perioperative period are also associated with the pathogenesis of POCD. They find that both POCD patients who underwent coronary artery bypass grafting and non-cardiac surgery had abnormally elevated levels of exosomal circRNA-089763, offering new candidate biomarkers for POCD (Wang et al., 2019; Zhou et al., 2020).

Postoperative delirium (POD) is a neuropsychiatric syndrome after surgical procedures, characterized by acute disturbances in consciousness, cognition, and emotional state. Approximately 15%–25% older adults aged 70 years and above will suffer from POD after major surgeries (Cho et al., 2024). In clinical practice, POD is primarily diagnosed through patient-reported symptoms, mental status evaluations, and clinical assessments (Oh and Park, 2019). Some researchers have identified significantly differentially expressed exosomal miRNAs between patients with and without POD, which may help identify individuals at risk of POD following surgery and clarify its pathogenesis (Cho et al., 2024). Xu and colleagues identified the miRNA profiles of serum exosomes from patients with and without POD, who had undergone total hip arthroplasty or total knee arthroplasty. They found that 16 miRNAs were significantly upregulated and 41 miRNAs were significantly downregulated in patients with POD compared to non-POD patients after miRNA sequencing and analysis, and suggested that these miRNA dysregulations play a key role in the pathogenesis of POD because they are involved in regulating neurotrophin signaling, neuroactive ligand-receptor interactions, and pathways that influence neuronal plasticity and cell viability. Specific exosomal miRNAs exhibit distinct alterations in patients who develop POD following surgery, indicating that they could serve as potential preoperative biomarkers for POD (Xu et al., 2025). Cho and colleagues tested exosomal miRNAs from the plasma of POD patients and non-POD patients aged 70 and above to find predictive POD biomarkers after spine surgery. They identified a total of 142 significantly differentially expressed miRNAs by using the miRNA profiling, which were associated with psychological/neurological disorders. Among these, 91 miRNAs were upregulated, and 51 miRNAs were downregulated in POD patients. Notably, changes in miR-548 can affect cognitive function, and four members of the miR-548 family (miR-548a-3p, miR-548ar-5p, miR-548j, and miR-548z) were upregulated in the patients with POD. Several other miRNAs linked with neurological and psychological disorders were also upregulated in POD patients. The researchers proposed that these differentially expressed miRNAs may function as upstream regulators of SPI1, AR, CDKN2A, and VEGFA, initiating POD onset before overt cognitive or mental symptoms emerge, and their alterations may reflect the early-stage process of POD development. Among them, six miRNAs, including miR-652-5p, miR-367-3p, miR-208a-3p, miR-548a-3p, miR-1246, and miR-520b, can serve as potential candidate miRNAs that can predict POD (Cho et al., 2024). In addition to miRNAs, Yan and colleagues conducted an integrated proteomic and metabolomic analysis of plasma exosomes from patients with and without POD. They found that significant differences in exosomal metabolites and proteins were closely related to potential metabolic pathways implicated in the development of POD, suggesting that other components of plasma exosomes can also serve as biomarker candidates for POD, such as MMP9, MPO, TLR2, ICAM1, and glutamate (Yan et al., 2024). Furthermore, exosomes can carry disease-specific cargos, for instance, α-synuclein (α-syn). α-syn is a neuronal protein associated with the pathogenesis of several forms of dementia. Since the plasma exosomal α-syn level was highly correlated with the α-syn content within the neuron, Yuan and colleagues measured plasma exosomal α-syn concentrations in patients with and without POD following hip fracture surgery. They found a positive correlation between changes in plasma exosomal α-syn concentration and delirium severity, suggesting that measuring exosomal α-syn may help identify older patients at risk for POD after hip fracture surgery (Yuan et al., 2020). Beak and colleagues identified biomarkers of POD from urinary exosomes using digital immunoassay technology. They found that Tau concentrations were significantly higher in urinary exosomes from patients with severe delirium than in those without delirium. Tau is already used as a biomarker of cognitive dysfunction. Analyzing urinary exosomes may help clinicians in accurately detecting delirium in older patients after spinal surgery (Baek et al., 2023).

### 3.2 Postoperative allograft rejection

Organ transplantation offers patients with end-stage organ dysfunction a renewed chance for survival. However, acute allograft rejection remains a leading cause of graft failure in the postoperative period. Acute rejection is primarily driven by T lymphocytes that recognize donor major histocompatibility complex (MHC) antigens, thereby triggering an adaptive immune response and a robust polyclonal inflammatory cascade (Marino et al., 2016). Tissue biopsy of transplanted organs is the gold standard for diagnosing solid organ allograft rejection. However, in addition to the risks of bleeding and damage to the allograft or surrounding structures, a biopsy cannot be dynamically detected. Over the past decade, the role of exosomes released by allografts and immune cells has gained increasing recognition in assessing graft function and immune acceptance or rejection (Gołębiewska et al., 2021). Analyzing the contents of these exosomes could facilitate the development of non-invasive biomarkers for diagnosing allograft rejection (Gonzalez-Nolasco et al., 2018; Romero-García et al., 2023; Saravanan et al., 2023). Currently, some studies have already demonstrated the potential value of exosomes for early postoperative monitoring of graft function and for diagnosing rejection (Romero-García et al., 2023).

Renal transplantation is currently the treatment of choice for end-stage kidney disease, but patients require long-term monitoring to detect allograft rejection. Recent studies have indicated that urinary exosomes may offer valuable diagnostic information for transplant rejection. El Fekih and colleagues isolated urinary exosomal mRNAs from 175 patients who underwent a clinically indicated kidney transplant biopsy and identified an exosomal mRNA signature that significantly correlates with kidney rejection using an SVM classifier. They further identified an additional gene signature (e.g., CXCL11, CD74, C3, IFNAR2, and CD44) from these exosomal mRNA signatures for the diagnosis of T cell–mediated rejection (TCMR) and/or antibody-mediated rejection (ABMR) in kidney transplants with high predictive values. Researchers suggested that urinary exosomal mRNAs are highly stable, and their mRNA signatures enable more accurate nucleic acid profiling, providing a powerful noninvasive screening tool for kidney allograft rejection (El Fekih et al., 2021). Seo and colleagues also identified 8 microRNAs of urinary exosomes as candidate biomarkers of acute rejection, including hsa-miR-4488, hsa-miR-4532, hsa-miR-21-5p, hsa-miR-155-5p, hsa-miR-210-3p, hsa-miR-223-3p, hsa-miR-31-5p, and hsa-miR-373-3p. They found that these distinctive expression signatures of urinary exosomal microRNAs could help differentiate AR from other allograft statuses. For example, a three-microRNA AR signature comprising hsa-miR-21-5p, hsa-miR-31-5p, and hsa-miR-4532 exhibited fair discriminative power in identifying acute rejection within the validation cohort (Seo et al., 2023).

Currently, endomyocardial biopsy (EMB) remains the clinical gold standard for distinguishing acute cellular rejection (ACR) and antibody-mediated rejection (AMR). But this invasive technique carries risks of complications and causes significant patient discomfort. Habertheuer and colleagues suggested that transplant heart exosome profiling offers a noninvasive, high-accuracy method for monitoring early acute rejection, based on their observation that transplanted hearts release donor-specific exosomes into the recipient’s circulation that are conditionally altered during immunologic rejection in a rodent heterotopic heart transplantation model (Habertheuer et al., 2018). Some scholars found that exosomes from the transplanted heart also express human leukocyte antigen class-I (HLA-I) molecules. In the patient who developed rejection, the exosomes contained C4d protein, a marker of AMR, indicating that further characterization of circulating donor heart exosomal cargoes may provide new insight for the diagnosis of cardiac allograft rejection (Hu et al., 2020). Castellani and colleagues investigated differences in plasma-derived exosome surface protein profiles between patients undergoing rejection and those with stable disease. They found that exosome concentrations were significantly increased in patients undergoing rejection, and their surface markers could serve as discriminants between stable patients and those with ACR or AMR. Compared to the exosomes of the patients with stable conditions, CD3, CD2, ROR1, SSEA-4, human leukocyte antigen (HLA)-I, and CD41b were increased in the exosomes of ACR patients, whereas HLA-II, CD326, CD19, CD25, CD20, ROR1, SSEA-4, HLA-I, and CD41b were increased in the exosomes of ANR patients. Based on these differential protein marker profiles, the researchers constructed a diagnostic model and validated it in an external cohort of patients to distinguish patients undergoing rejection from those without. This study highlighted the diagnostic potential of circulating exosomes as biomarkers for monitoring cardiac allograft rejection (Castellani et al., 2020).

In lung transplantation research, Habertheuer and colleagues observed changes in the circulating exosome profiles before acute rejection occurred in rat orthotopic lung transplant models. They transplanted left lungs from Wistar transgenic rats expressing human CD63-GFP into recipient animals and then collected plasma exosomes to detect the exosomes expressing human CD63-GFP. The results showed that a decrease in peripheral donor exosome levels before histological evidence of acute rejection in grafts, indicating that the real-time monitoring of circulating exosome profiles contributes to the diagnosis of acute rejection (Habertheuer et al., 2022). Gunasekaran and colleagues provided clinical evidence supporting the use of exosomes for diagnosing lung transplantation rejection. They isolated exosomes from serum and bronchoalveolar lavage fluid of 30 lung transplant recipients (LTxRs) who had no evidence of rejection or microbial infection (stable patients) or who had acute rejection (AR) or bronchiolitis obliterans syndrome (BOS). Their analysis revealed that lung-associated self-antigens (SAgs) and donor HLA were present on the surface of exosomes from patients with AR and BOS, but not on those from stable patients, suggesting that these exosomes could serve as noninvasive biomarkers of impending rejection. In addition to the SAgs Col-V and Kα1T, exosomal miRNAs were differentially expressed in the serum of patients with BOS or AR compared to stable recipients. In particular, specific miRNAs involved in inflammation (miR-182), endothelial activation (miR-92a), antibody-mediated chronic rejection (miR-142-5p), and T Cell activation (miR-155) may contribute to the pathogenesis of AR and BOS. Notably, these exosomes, originating from the transplanted lung, appear in the circulation and at the local site and can be detected before the clinical diagnosis of AR or BOS (Gunasekaran et a., 2017). In a subsequent study, the same researchers further discovered that exosomes from the LTxRs patients with BOS contain surface expression of donor HLA, lung SAg, MHC-II, costimulatory molecules, cell adhesion molecules, and various transcription factors, which contribute to stimulate T-cell responses by direct, semi-direct, and indirect pathways of SAg presentation, thereby triggering immune responses against both allo and lung SAg. Blocking exosome production and release may represent a viable strategy for preventing chronic rejection, suggesting that exosomes may also be therapeutic targets in addition to their predictive role (Gunasekaran et al., 2018). Recently, some scholars discussed the triple role of exosomes in lung transplantation, including a) using exosomes as a biomarker for rejection after lung transplantation, b) the mechanism of exosome-mediated activation of the immune response, and c) the potential of exosomes as a therapeutic strategy, offering insights for future therapies and biomarker identification to improve patient outcomes (Rao et al., 2025).

### 3.3 Postoperative septic shock

Postoperative septic shock (PSS) is a life-threatening complication. Survey data indicate that the incidence of septic shock after hip fracture surgery was only 0.6%. Still, the 30-day mortality rate of these patients with septic shock was 40.8%, which is higher than the 16.2% of patients with sepsis (Gonzalez et al., 2023). Early identification of septic shock is critical for reducing postoperative mortality. Distinguishing septic shock from non-septic shock remains challenging due to their overlapping signs and symptoms. Previous studies have attempted to use the preoperative/postoperative white blood cell ratio or identify differentially expressed genes to distinguish septic shock from non-septic shock in postsurgical patients, aiming to avoid unnecessary broad-spectrum antibiotic use and the associated risks of antimicrobial resistance, secondary infections, and rising healthcare costs (Martínez-Paz et al., 2021; Hao et al., 2022). The roles of exosomes in the pathogenesis of sepsis have attracted attention, especially the changes in their content and function during sepsis (Murao et al., 2020; Gong et al., 2024). Exosomal proteins and miRNAs offer a promising source of biomarkers for sepsis and sepsis-related organ failure (Yuan et al., 2024). Recently, García-Concejo and colleagues conducted a multicentre, prospective study to identify exosomal miRNA signatures to differentiate septic shock from non-septic shock in postsurgical patients. They collected plasma samples from postoperative patients within 24 h of shock diagnosis and conducted exosomal miRNA sequencing. A total of 30 miRNAs show a significant difference between septic and non-septic shock patients. Among them, six miRNAs demonstrated strong diagnostic capabilities for septic shock, such as miR-100-5p, miR-484, miR-10a-5p, miR-148a-3p, miR-342-3p, and miR-451a. Notably, the combination of three specific miRNAs (miR-100-5p, miR-148a-3p, and miR-451a) demonstrated strong promise as complementary and reliable biomarkers for accurately diagnosing sepsis in post-surgical patients experiencing shock, with their validity confirmed through qPCR analysis (García-Concejo et al., 2025).

### 3.4 Postoperative atrial fibrillation

Postoperative atrial fibrillation (POAF) is the most common perioperative cardiac arrhythmia following cardiovascular and non-cardiac thoracic surgery (Bjerrum et al., 2020). POAF typically manifests 2–4 days after surgery, with episodes that are often transient and self-limiting, yet it can cause hemodynamic instability, prolong hospitalization, and increase mortality (Dobrev et al., 2019). Previous studies have explored potential links between exosomes and atrial fibrillation to improve diagnosis and prognosis. Compared with other components in exosomes (e.g., mitochondrial DNA and protein), exosomal miRNAs have great potential for diagnosing atrial fibrillation (Huang et al., 2021; Xiang et al., 2022). Bai and colleagues identified 23 differentially expressed miRNAs in plasma exosomes of POAF patients and non-POAF patients who underwent elective coronary artery bypass grafting (CABG) surgery. Among the five miRNAs found to be significantly altered, only miR-122-5p was up-regulated in the POAF patient, and its target genes were involved in multiple pathways related to cardiac function, including the NF-kappa B signaling pathway, toll-like receptor signaling pathway, and oxidative stress-induced intrinsic apoptotic signaling pathway. Notably, the ROC curve demonstrated that miR-122-5p exhibited high sensitivity and specificity for diagnosing POAF, indicating that exosomal miR-122-5p can serve as a novel biomarker for assessing disease severity or prognosis, thereby facilitating risk stratification, individualized therapeutic strategies, drug intervention, and POAF evaluation (Bai et al., 2022). A recent study identified 51 differentially expressed proteins in plasma exosomes from POAF patients and non-POAF patients following CABG surgery. The researchers found that abnormally expressed plasma amyloid P-component and Collagen alpha-2(I) chain in plasma exosomes correlate with POAF, suggesting that exosomal proteins represent potential diagnostic biomarkers for POAF and warrant further investigation (Chen et al., 2026).

### 3.5 Postoperative sleep disturbance

Postoperative sleep disturbance (PSD) is a common but often overlooked complication following surgery, with an incidence ranging from 30% to 80%. The clinical manifestations of PSD include reduced total sleep time, increased frequency of awakenings, decreased sleep quality, and frequent nightmares (Jian et al., 2025; Wu et al., 2025). PSD has been associated with increased risks of acute postoperative pain, postoperative delirium, and postoperative cardiovascular events (Fadayomi et al., 2018; Liu and Tang, 2025). Therefore, early recognition and intervention for PSD are critical to improving patient outcomes after surgery. Circadian rhythms are susceptible to disruption by altered sleep patterns, which in turn influence the biogenesis, secretion, and cargo selection of exosomes (Tao and Guo, 2018; Yeung et al., 2022; Smith, 2025). Khalyfa and colleagues observed changes in plasma exosome cargo and metabolic function in a mouse model of chronic nocturnal shift work, particularly in the regulation of canonical clock genes, including BMAL1, CRY2, and PER1 (Khalyfa et al., 2017). They subsequently investigated the effect of circulating exosomal miRNAs, as messengers of circadian misalignment to peripheral tissues, on metabolic dysfunction in individuals with altered sleep patterns. They found significant differences in plasma exosomal hsa-mir-3614-5p, which regulates circadian clock-related genes, between night-shift and day-shift workers, suggesting that exosomal miRNAs are involved in disruptions of sleep in circadian rhythm (Khalyfa et al., 2020). Given the close relationship between exosomes and sleep disturbance, some scholars have discussed the roles of exosomes and their miRNAs in postoperative sleep disturbance (Gu and Zhu, 2021). However, high-quality reviews are still needed to provide accurate and valuable information for this field. Meanwhile, further investigation is required to examine the status of exosomes and their miRNA changes in PSD for early diagnosis and prevention.

## 4 Challenges and limitations

The above research report confirms that exosomes may serve as novel biomarkers for predicting surgical complications, offering an alternative to traditional blood-based biomarkers (Table 1). Despite promising prospects, several challenges hinder the development and clinical translation of exosomal biomarkers. First, heterogeneity presents a major obstacle that must be addressed. Researchers can use existing detection methods to identify similarities and differences in the specific contents of exosomes secreted by the same tissue cells under both physiological and pathological conditions, and further reveal the key role of exosomes in disease pathogenesis. However, the cargo-sorting mechanism remains poorly understood, which currently prevents us from explaining why certain contents appear in the released exosomes. Intracellular hypoxia, calcium concentration, and pH influence both exosome generation and release, thereby affecting their yield (Savina et al., 2003; Zhao et al., 2017; Jiang H. et al., 2022). Especially perioperative management, anesthesia procedures, and surgical strikes can cause significant changes in the body’s internal environmental state. In addition to the above internal environmental factors, other variables may affect exosome biogenesis, including age, gender, ethnicity, environmental factors, and pre-existing conditions. Therefore, even the same postoperative complication may exhibit different biomarkers depending on the surgical method, as shown in Table 1. Second, exosomes are present in various body fluids, facilitating their collection for assessing organ or system status. Most studies obtain exosomes from peripheral blood. However, blood cells also secrete exosomes or exosome-like vesicles, complicating the isolation of target-tissue-derived exosomes. Urinary exosomes may serve as ideal biomarkers related to kidney disease. However, they can be contaminated by immune cells, bacteria, and yeast from the urogenital tract (Zhou et al., 2023). Therefore, standard operating procedures are required for the selection, collection, preparation, storage, and transportation of body fluid samples. Therefore, standard operating procedures are required for the selection, collection, preparation, storage, and transportation of body fluid samples. However, the studies reviewed here have not yet fully addressed this issue. Third, there is currently no consensus on methods for isolating and purifying exosomes. Some scholars have compared the efficiency of isolation methods, and although a particular approach may prove optimal in one context, no single method suits all studies. Rather, the choice of method depends on the sample, subtypes, size, and budget (Veerman et al., 2021; Gao et al., 2022; Ansari et al., 2024), which may lead to variation in exosomal cargo abundance and cause bias in content analysis. The time and cost involved are also important factors to consider when selecting methods. In addition to an efficient extraction method, highly sensitive techniques for analyzing exosomal contents are needed. The summarized studies in this review employ techniques based on distinct principles, yielding varying levels of purity and quantity, and use analysis techniques that may critically affect subsequent analyses. Fourth, existing studies have identified significant differences in the expression levels of several exosomal components between patients with and without postoperative complications, indicating their potential diagnostic value. However, it remains unclear which of these components holds sufficient diagnostic utility to serve as a reliable biomarker for postoperative complications. Thus, more detailed, large-scale clinical comparative studies are needed in the future.

**TABLE 1:** Literature examples of clinical studies on using exosomes as a biomarker in postoperative complications.

Postoperative complications	Surgery	Exosome source	Isolation methods	Detection indicators	Potential biomarkers	References
Postoperative cognitive dysfunction	Hip replacement surgery	Plasma	Ultrafiltration	Exosomal miRNAs	miR-584-5p	Zhao et al., (2022)
	Laparoscopic cholecystectomy	Serum	Not mentioned	Exosomal lncRNAs	HOTAIR, GAS5, BLACAT1	Campo et al. (2024)
	Non-cardiac surgery	Plasma	Not mentioned	Exosomal circ RNAs	circRNA-089763	Zhou et al. (2020)
	Coronary artery bypass grafting surgery	Plasma	Ultrafiltration	Exosomal circ RNAs	circRNA-089763	Wang et al. (2019)
Postoperative delirium	Total hip/knee arthroplasty	Plasma	Ultrafiltration	Exosomal miRNAs	has-miR-1299, has-miR-320a-3p, has-miR-140-3p, has-miR-1288-5p, has-miR-210-3p, has-miR-142-3p, et al.	Xu et al. (2025)
	Spine surgery	Plasma	Polymer precipitation (ExoQuick)	Exosomal miRNAs	miR-652-5p, miR-367-3p, miR-208a-3p, miR-548a-3p, miR-1246, and miR-520b	Cho et al. (2024)
	Cardiopulmonary bypass and coronary artery bypass grafting surgery	Plasma	Immunoaffinity capture (EV-trap)	Exosomal proteins/metabolites	MMP9, TLR2, ICAM1, S100B, and glutamate	Yan et al. (2024)
	Hip fracture surgery	Plasma	Immunoaffinity capture (Dynabeads® Antibody Coupling Kit)	Exosomal proteins	α-synuclein	Yuan et al. (2020)
	Spine surgery	Urine	Digital immunoassay technology	Exosomal proteins	Tau, ubiquitin carboxy-terminal hydrolase L1	Baek et al. (2023)
Postoperative allograft rejection	Renal transplantation	Urine	Urine-exosome isolation kit	Exosomal mRNAs	CD74, C3, CXCL11, CD44, and IFNAR2	El Fekih et al. (2021)
	Renal transplantation	Urine	Spin column-based exoRNeasy serum/plasma midi kits	Exosomal miRNAs	hsa-miR-4488, hsa-miR-4532, hsa-miR-21-5p, hsa-miR-155-5p, hsa-miR-210-3p, hsa-miR-223-3p, hsa-miR-31-5p, and hsa-miR-373-3p	Seo et al. (2023)
	Heart transplantation	Plasma	Immunoaffinity capture	Exosomal proteins	HLA-I, CD2, SSEA-4, ROR1, SSEA-4, HLA II, and CD41b	Habertheuer et al. (2018)
	Lung transplantation	Serum/Bronchoalveolar lavage fluid	Ultracentrifugation/Polymer precipitation/Density-gradient ultracentrifugation	Exosomal proteins/miRNAs	SAgs, miR-92a, miR-182, miR-142-5p, and miR-155	Gunasekaran et al. (2017)
Postoperative septic shock	Any type of surgery	Plasma	Size-exclusion chromatography	Exosomal miRNAs	miR-100-5p, miR-484, miR-10a-5p, miR-148a-3p, miR-342-3p, and miR-451a	García-Concejo et al. (2025)
Postoperative atrial fibrillation	Coronary artery bypass grafting surgery	Plasma	Ultrafiltration/Polymer precipitation	Exosomal miRNAs	miR-122-5p	Bai et al. (2022)
	Coronary artery bypass grafting surgery	Plasma	Exosome extraction and purification kit (Umibio)	Exosomal proteins	Serum amyloid P-component (SAP) and collagen alpha-2(I) chain	Chen et al. (2026)

## 5 Conclusion

Recent evidence indicates that exosomes are promising non-invasive biomarkers for early diagnosis and disease monitoring in cancers, cardiovascular diseases, and neurodegenerative disorders, providing a less invasive and more accessible approach for clinical screening and tracking disease progression. The research findings presented in this review demonstrate that exosomes also hold practical value in predicting postoperative complications. Profiles of exosomal proteins and nucleic acids offer a comprehensive view of disease status that conventional methods often struggle to capture. Although challenges remain in the clinical translation of exosome-based diagnostics, exosomes hold potential for application in the prevention and management of postoperative complications and represent an emerging avenue in exploring biomarkers. As research on exosomes continues to advance and extraction and testing techniques are further refined and integrated with bioinformatics or machine learning (ML) techniques, exosome-based diagnosis of postoperative complications will attract greater attention and undergo further development.
